# The antibacterial activity of extracts of nine plant species with good activity against *Escherichia coli* against five other bacteria and cytotoxicity of extracts

**DOI:** 10.1186/s12906-017-1645-z

**Published:** 2017-02-28

**Authors:** Ishaku Leo Elisha, Francien S. Botha, Lyndy Joy McGaw, Jacobus Nicolaas Eloff

**Affiliations:** 1 Phytomedicine Programme, Department of Paraclinical Sciences, Faculty of Veterinary Science, University of Pretoria, Private Bag X04, Onderstepoort, 0110 Pretoria, South Africa; 2 Present Address: Drug Development Section, Biochemistry Division, National Veterinary Research Institute, P.M.B. 01 Vom, Plateau State Nigeria

**Keywords:** Antibacterial activity, Potency, Efficacy, Cellular safety, Nosocomial bacteria, Correlation

## Abstract

**Background:**

The development of antibiotic resistant bacteria stems from a number of factors, including inappropriate use of antibiotics in human and animal health and their prolonged use as growth promoters at sub-clinical doses in poultry and livestock production. We were interested in investigating plants that could be useful in protecting humans or animals against diarrhoea. We decided to work on extracts of nine plant species with good activity against *Escherichia coli* based on earlier work in the Phytomedicine Programme. Leaves of nine medicinal plant species with high antibacterial activity against *Escherichia coli* were extracted with acetone and their minimal inhibitory concentration (MIC) values determined using a microplate serial dilution technique against Gram-positive (*Staphylococcus aureus*, *Enterococcus faecalis* and *Bacillus cereus*) and Gram-negative (*Escherichia coli*, *Salmonella* Typhimurium and *Pseudomonas aeruginosa*) bacteria. Bioautography was used to determine the number of bioactive compounds in each extract. In vitro safety of the extracts was determined using the 3-(4,5-dimethylthiazolyl-2)-2,5-diphenyltetrazolium bromide reduction assay on Vero cells.

**Results:**

The extracts were active against all the pathogens with average MICs ranging from 0.02 to 0.52 mg/ml. As expected *E. coli* was relatively sensitive, while *E. faecalis* and *S.* Typhimurium were more resistant to the extracts (average MICs of 0.28 mg/ml and 0.22 mg/ml respectively). *Cremaspora triflora* and *Maesa lanceolata* leaf extracts had higher activity than the other extracts against Gram-positive and Gram-negative pathogens with mean MICs of 0.07 mg/ml and 0.09 mg/ml respectively. Extracts of *Maesa lanceolata* and *Hypericum roeperianum* had the highest total antibacterial activity (TAA) at 1417 and 963 ml/g respectively. All extracts with the exception of that of *Maesa lanceolata*, *Elaeodendron croceum* and *Calpurnia aurea* had relatively low cytotoxicity with LC_50_ > 20 μg/ml. *Cremaspora triflora* had the best selectivity index (SI) against *S. aureus* and *E. coli* of 2.87 and 1.15 respectively. *Hypericum roeperianum* had a SI of 1.10 against *B. cereus*. Bioautography revealed 1–6 visible antimicrobial compounds that were generally non-polar.

**Conclusions:**

There was a weak positive, but statistically non-significant correlation between the potency of the extracts and their cytotoxicity (*R* = 0.45, *ρ* > 0.05). The activity of the extracts on the test bacteria was in some cases not correlated with cytotoxicity, as shown by selectivity indices >1. This means that cellular toxicity was probably not due to compounds with antibacterial activity. Some of the extracts had a good potential for therapeutic use against the bacterial pathogens or for application in treating diarhoea. It does not appear that activity against *E. coli* is a good predictor of activity against Gram-negative rather than Gram-positive bacteria. Further investigation is in progress on *C. triflora* and *H. roeperianum*, both of which had promising activities and potential safety based on cytotoxicity.

## Background

Rising antibiotic resistanceand the scarcity of new antimicrobialshas long beenacknowledged [1, 2]. A major challenge in global health care is the need for novel, effective and affordable medicines to treatmicrobial infections, especially in developing countries of the world, where up to one-halfof deathsare due to infectious diseases [3, 4].

Some *Staphylococcus* spp. and *Streptococcus* spp. involved in the pathogenesis of respiratory and skin infections, along with Pseudomonads and members of the Enterobacteriaceae causing gastrointestinal, urogenital diseases and wound contamination are resistant to virtually all of the older antibiotics [5]. Clinical isolates of *Staphylococcus aureus*, the leading cause of nosocomial infections, are increasingly resistant to an array of antimicrobial agents like penicillin, gentamicin, tobramycin, amikacin, ciprofloxacin, clindamycin, erythromycin, chloramphenicol, trimethoprim-sulfamethoxazole and vancomycin [6].

The development of antimicrobial-resistant bacterial species stems from a number of factors which include the prevalent and sometimes inappropriate use of antibiotics, extensive use of these agents as growth enhancers in animal feed, and increased transboundary passage of antibiotic-resistant bacteria [6]. The problem of antibiotic resistance in humans and animals will continue for a long time [7]. Against this backdrop, the development of alternative drug classes to treat such infectious diseases is urgently required [4].

Plants have an amazing ability to produce a wide variety of secondary metabolites, like alkaloids, glycosides, terpenoids, saponins, steroids, flavonoids, tannins, quinones and coumarins [8]. These biomolecules are the source of plant-derived antimicrobial substances (PDAms) [4]. Some natural products are highly efficient in the treatment of bacterial infections [9].

South Africa has a large diversity of plant species containing many useful bioactive constituents [10, 11]. The lack of access to Western primary health care and veterinary services in many rural parts of the world has helped sustain the use of traditional medicine to treat both humans and animals. Even where orthodox medicines are readily available, a large percentage of the population still use herbal remedies along with or in preference to conventional medicines [12].

We are interested in applying plant extracts to treat diarhoea in humans and animals. Because *E. coli* plays an important role in causing diarrhoea, we selected nine species (*Hypericum roeperianum, Cremaspora triflora, Heteromorpha arborescens, Pittosporum viridiflorum, Bolusanthus speciosus*, *Calpurnia aurea, Maesa lanceolata, Elaeodendron croceum* and *Morus mesozygia*) from the Phytomedicine database of the University of Pretoria that had good activity against *E. coli* [13]. Traditional use was not taken into aaccount in selecting these species. Here we discuss the in vitro antibacterial activity of acetone leaf extracts of these nine different South African plant species against three Gram-positive and three Gram-negative bacteria (*Staphylococcus aureus, Enterococcus faecalis, Bacillus cereus, Pseudomonas aeruginosa, Salmonella* Typhimurium*,* and *Escherichia coli*). We determined the number of antibacterial compounds present in extracts by bioautography and the correlation between cytotoxicity and potency against Gram-negative and Gram-positive bacteria. We also determined whether extracts with high activity against Gram-negative bacteria would have higher activity against other Gram-negative bacteria than against Gram-positive bacteria.

## Methods

### Collection of plant material, drying and storage

The leaves of *Hypericum roeperianum* G.W. Schimp.exA.Rich. var. *Roeperianum,* (Hypericaceae, PRU 120126)*, Cremaspora triflora* (Thonn.) K.Schum (Rubiaceae, PRU 120129)*, Heteromorpha arborescens* (Spreng.) Chan. &Schltdl (Apiaceae, PRU 120026)*, Pittosporum viridiflorum* Sims (Pittosporaceae, PRU 120025)*, Bolusanthus speciosus* (H. Bolus) Harms (Fabaceae, PRU 120027)*, Calpurnia aurea* (Aiton) Benth ssp aurea (Fabaceae, PRU 120125)*, Maesa lanceolata* Forssk (Maesaceae PRU120125)*, Elaeodendron croceum* (Thunb.) DC (Celastraceae, PRU 120127) and *Morus mesozygia* Stapf ex A.Chev (Moraceae, PRU 120128) were collected in the summer of 2013, at the University of Pretoria Botanical Garden, Pretoria National Botanical Garden and Lowveld National Botanical Garden in Nelspruit, Mpumalanga Province South Africa. Voucher specimens were prepared and deposited in the HGWJ Schweickerdt Herbarium of the University of Pretoria (PRU).

Methods developed in the Phytomedicine Programme were used [13]. Leaves were examined and those attacked by insects or microbes were removed. Harvested leaves were stored in open mesh loosely woven bags to ensure airflow for quick drying indoors at room temperature to minimise chemical changes by microbial attack after collection. The leaves were ground to a fine powder using a Jankel and Künkel Model A10 mill. The powder was stored in tightly closed glass containers in the dark at room temperature.

### Extraction

Acetone (technical grade, Merck) was used as an extractant in the assays using a ratio of 1:10 of leaf material to extractant. Acetone is the best choice as an extractant mainly due to its ability to extract compounds of a wide range of polarities [14, 15], its low toxicity to bioassay systems [16] and because it is easy to remove from extracts. Three grams (3.0 g) of each tree leaf sample was extracted with 30 ml acetone. The resulting suspension was shaken vigorously in 50 ml polyester centrifuge tubes and centrifuged at 4000 x g for 10 min (Hettich Centrifuge, Rotofix 32A, Labotec, Johannesburg, South Africa). The supernatants were decanted into preweighed glass vials through Whatman No. 1 filter paper and concentrated to dryness under a stream of cold air. The dried extracts were made up to a concentration of 10 mg/ml (stock solution) in acetone to be used in subsequent assays and stored at 5 °C in tightly stoppered glass tubes.

### Thin layer chromatography (TLC) analysis of the extracts

Three solvent systems with differing polarities were used to analyse 100 μg of the extract placed in a band of 1 cm by thin layer chromatography (Merck aluminium-backed plates, silica gel 60 F_254_) [14]. These were benzene: ethanol: ammonium hydroxide (90:10:1, BEA, non-polar basic), chloroformethylacetate: formic acid (5:4:1, CEF, intermediate polarity, acidic) and ethylacetate: methanol: water (40:5.4:5, EMW, polar, neutral). Visible bands were marked under daylight and ultraviolet light (254 nm and 360 nm wavelengths, Camac universal UV light lamp TL-600) before spraying with freshly prepared vanillin (0.1 g vanillin, 28 ml methanol, 1 ml sulphuric acid) spray reagent [17]. The plates were carefully heated at 105 °C for optimal colour development.

### Test organisms

Microorganisms used in this study represent pathogenic species commonly associated with nosocomial infections. The bacteria were maintained in the Phytomedicine Laboratory at Onderstepoort, University of Pretoria and consisted of three Gram-positive strains, *E. faecalis* (ATCC 29212), *B. cereus* (ATCC 21366) and *S. aureus* (ATCC 29213), and three Gram-negative strains, *E. coli* (ATCC 25922), *S.* Typhimurium (ATCC 39183) and *P. aeruginosa* (ATCC 27853). All the bacterial strains were subcultured from the original culture, stored at −70 °C and maintained on Müller-Hinton (MH) agar plates at 4 °C, and grown at 37 °C when required. The strains used are those recommended by the United States National Committee for Clinical Laboratory Standardsto compare antibiotics [18].

### Qualitative antibacterial activity assay by bioautography

The bioautography procedure described by Begue and Kline [19] was used to determine the number of antimicrobial compounds separated by TLC. Thin layer chromatography plates were prepared and developed in the different solvent systems, dried overnight under a stream of air to remove residual solvent, which might inhibit bacterial growth. One of the plates was sprayed with vanillin spray reagent and the others with two bacterial cultures –*S. aureus* (ATCC 29213) and *E. coli* (ATCC 25922). These bacterial species are the major cause of nosocomial infections in the hospitals [20]. Ten ml of a dense fresh bacterial culture was centrifuged at 4000 x g for 15 min to concentrate the bacteria. The supernatant was discarded and the combined pellets re-suspended in 2–4 ml of fresh Müller-Hinton broth. The plates were sprayed with the concentrated suspension until they were just wet, dried in air to remove excess liquid and incubated overnight at 37 °C at 100% relative humidity. After incubation, plates were sprayed with a 2 mg/ml solution of *p*-iodonitrotetrazolium violet (Sigma chemicals). Clear zones on the chromatograms indicated inhibition of growth by separated antimicrobial compounds after incubating for about 1–2 h at 37% under 100% relative humidity [19].

### Quantitative antibacterial activity assay by minimum inhibitory (MIC) and total activity

A widely accepted sensitive serial dilution microplate method [21] was used to determine the minimum inhibitory concentration (MIC) of the plant extracts against six bacterial strains in triplicate. This biological assay was chosen because of its simplicity, reproducibility, sensitivity, and relatively low cost while being a rapid method at the same time. Bacterial cultures grown overnight were adjusted to McFarland standard 1, equivalent to 3.0 x 10^8^ cfu/ml (*Staphylococcus aureus*), 2.1 x10^8^cfu/ml (*Enterococcus faecalis*), 1.3 x 10^8^ cfu/ml (*Bacillus cereus*), 3.7 x 10^8^ cfu/ml (*Escherichia coli*), 3.5 x 10^8^ cfu/ml (*Salmonella typhimurium*) and 3.2 x 10^8^ cfu/ml (*Pseudomonas aeruginosa*). The dried extracts were dissolved in acetone to a concentration of 10 mg/ml and 100 μl was added to the first well of a 96-well microtitre plate and serially diluted 1:1 with water. Bacterial cultures (100 μl) were added to each well. Starting with an extract concentration of 10 mg/ml, the bacteria were therefore subjected to final concentrations of 2.5 to 0.02 mg/ml.Gentamicin was used as positive control and acetone was used as asolvent control. The highest concentration the bacteria were subjected to was 25% acetone in the first well and decreased two-fold in each subsequent well. The growth of bacteria has never been inhibited by 25% acetone. Acetone had an MIC of 51% against six fungi followed by dimethylsulphoxide (45%), methanol (32%) and ethanol (30%) [16].

The microplates were incubated overnight at 37 °C in 100% relative humidity. As an indicator of growth, 40 μl of 0.2 mg/ml INT (p-iodonitrotetrazolium violet, Sigma®) dissolved in hot water was then added to the microplate wells and incubated at 37 °C for 2 h. The MIC was determined visually as the lowest concentration that led to growth inhibition after c. 2 h.

### Cytotoxic activity

The cytotoxicity of the acetone extracts against Vero monkey kidney cells was determined by using the 3-(4,5-dimethylthiazol-2-yl)-2, 5-diphenyltetrazolium bromide (MTT) reduction assay as previously described by Mosmann [22] with slight modifications. Cells were seeded at a density of 1 x 10^5^ cells/ml (100 μl) in 96-well microtitre plates and incubated at 37 °C and 5% CO_2_ in a humidified environment. After overnight incubation, 100 μl each of varying extract concentrations were added to the wells containing cells. Doxorubicin was used as a positive reference. A suitable blank control with equivalent concentrations of acetone was also included and the plates were incubated for 48 h in a CO_2_ incubator. Thereafter, the medium in each well was aspirated from the cells, cells were washed with PBS, and finally 200 μl fresh media was added to each well. Thirty microlitres of MTT (5 mg/ml in PBS) was added to each well and the plates were incubated at 37 °C for 4 h. The medium was aspirated from the wells and DMSO was added to solubilise the formazan crystals. The absorbance was measured using a BioTek Synergy microplate reader at 570 nm. The percentage of cell growth inhibition was calculated based on a comparison with untreated cells. The selectivity index values were calculated by dividing cytotoxicity LC_50_ values by the MIC values of the test bacteria in the same units (SI = LC_50_/MIC).

### Statistical analysis

The experimental results were expressed as mean ± standard deviation (SD) of three replicates. Where applicable, the data were subjected to one-way analysis of variance (ANOVA) and differences between samples were determined by two-tailed *t*-test after Bonferroni error correction of the predictive value. *P* values less than 0.05 were considered statistically significant. Microsoft Excel 2010 statistical package was used for all analyses.

## Results and discussion

### Plant extracts yield

Acetone extraction gave different extraction yields. *Hypericum roeperianum* had the highest yield (12%), followed by *Maesa lanceolata* (11.12%). The lowest extraction yield was obtained with *Morus mesozygia* 1.85% (Table 1). These yields were more or less in line with extract yields from 27 Combretaceae species [23]. Extraction yield from a plant has a great effect on the overall efficacy and selection for bioprospecting and in the calculation of total activity [24].

**Table 1:** Extract yield, Minimal Inhibitory Concentration (MIC) with standard deviation and Total Antibacterial Activity (TAA) of the nine selected acetone leaf extracts against Gram-negative test bacteria

		*Escherichia coli*		*Salmonella* Typhimurium		*Pseudomonas aeruginosa*	
Plants	% yield	MIC (mg/ml)	TAA (ml/g)	MIC (mg/ml)	TAA (ml/g)	MIC (mg/ml)	TAA (ml/g)
*Hypericum roeperianum*	12.0	0.13 ± 0.04	922.82	0.26 ± 0.07	461.41	0.08	1499.58
*Cremaspora triflora*	2.02	0.05 ± 0.02	403.33	0.32 ± 0.25	63.02	0.16	126.04
*Heteromorpha arborescens*	2.60	0.18 ± 0.10	144.63	0.31 ± 0.00	83.98	0.16	162.71
*Pittosporum viridiflorum*	2.72	0.11 ± 0.04	246.97	0.22 ± 0.13	123.48	0.16	169.79
*Bolusanthus speciosus*	2.30	0.08 ± 0.00	287.92	0.13 ± 0.04	177.18	0.16	143.96
*Calpurnia aurea*	2.86	0.04 ± 0.00	715.83	0.13 ± 0.04	220.26	0.16	178.96
*Maesa lanceolata*	11.12	0.04 ± 0.00	2780.83	0.16 ± 0.00	695.21	0.08	1390.42
*Elaeodendron croceum*	9.00	0.11 ± 0.04	817.88	0.26 ± 0.07	346.03	0.08	1124.58
*Morus mesozygia*	1.85	0.07 ± 0.02	263.81	0.16 ± 0.11	115.42	0.08	230.83
Gentamicin	NA	0.0008	NA	0.0002	NA	0.0002	NA
Average for extracts	NA	0.09 ± 0.04	NA	0.22 ± 0.07	NA	0.12 ± 0.04	NA

*NA* not applicable

### Bioautography

**Fig. 1:**
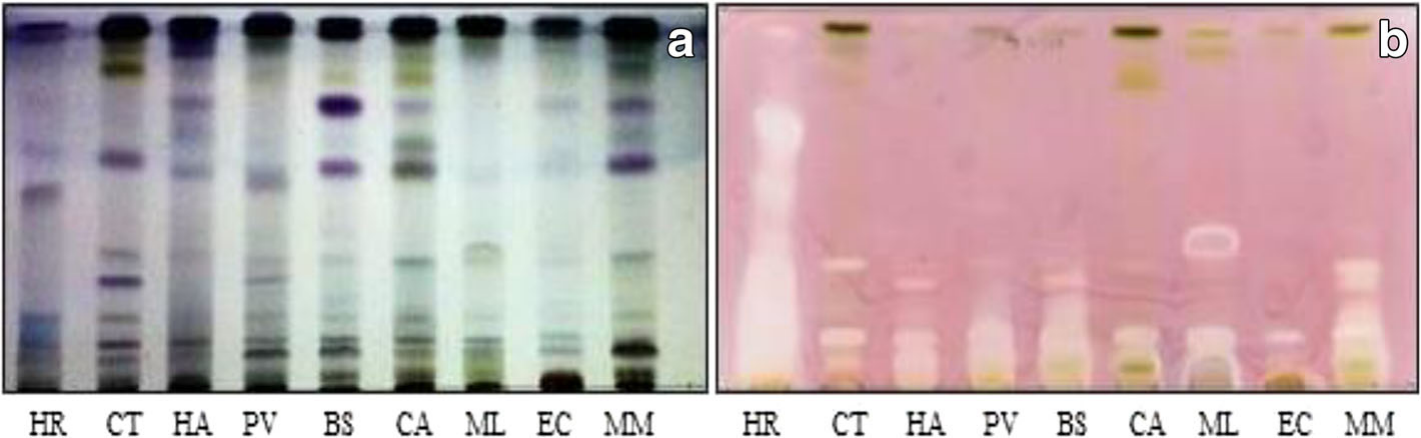
**a** Chromatogram developed in Benzene: Ethanol: Ammonia (BEA) solvent system of the different plant leaf acetone extracts sprayed with vanillin. **b** Bioautography of *Staphylococcus aureus* developed with BEA; *white* bands indicate compounds that inhibit the growth of the bacteria. HR = *Hypericum roeperianum*, CT = *Cremaspora triflora*, HA = *Heteromorpha arborescens*, PV = *Pittosporum viridiflorum*, BS = *Bolusanthus speciosus,* CA = *Calpurnia aurea*, ML = *Maesa lanceolata*, EC = *Elaeodendron croceum*, MM = *Morus mesozygia*

The non-polar BEA solvent system separated the active bands against *S. aureus* better than the other solvent systems, indicating that the bioactive compounds are relatively non-polar. The retention factor (R_f_), was calculated by dividing the distance moved by the compound by the distance of the solvent front. This represents the position of antimicrobial constituents on the chromatogram [25]. The highest number of active bands (6) against *S. aureus* bioautography was present in the in *H. roeperianum* (R_f_ = 0.12, 0.27, 0.38, 0.52, 0.67, 0.94) and *M. mesozygia* extracts (R_f_ = 0.09, 0.12, 0.16, 0.24, 0.29 and 0.32) respectively (Fig. 1). *Elaeodendron croceum* and *C. aurea* had only one active band against *S. aureus*. An antimicrobial compound with R_f_ value 0.12 was present in all of the plant extracts, indicating that the same compound is probably present in all the extracts (Fig. 1).

### Minimal inhibitory concentration and total antibacterial activity

**Fig. 2:**
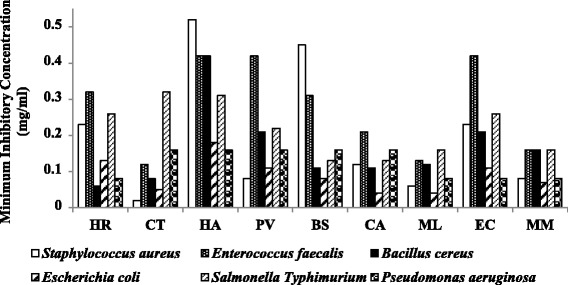
Average MIC values of the nine-acetone leaf extracts against all the test bacteria; the lower the MIC values the most potent the extract. There is a significant difference between the MIC values of the different crude extracts against the test bacteria (*ρ* < 0.05). HR = *Hypericum roeperianum*, CT = *Cremaspora triflora*, HA = *Heteromorpha arborescens*, PV = *Pittosporum viridiflorum*, BS = *Bolusanthus speciosus,* CA = *Calpurnia aurea*, ML = *Maesa lanceolata*, EC = *Elaeodendron croceum*, MM = *Morus mesozygia*

**Fig. 3:**
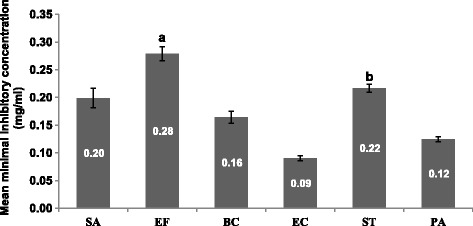
The mean MIC in mg/ml of the acetone leaf extracts of the nine plants against five different bacteria. SA = *Staphylococcus aureus*, EF = *Enterococcus faecalis*, BC = *Bacillus cereus*, EC = *Escherichia coli*, ST = *Salmonella* Typhimurium, PA = *Pseudomonas aeruginosa*, ^a^ = *Enterococcus faecalis* and *Escherichia coli* (*ρ* < 0.01), ^b^ = *Salmonella* Typhimurium and *Escherichia coli* (*ρ* < 0.01)

The serial microdilution results were analysed using the Analysis of Variance (ANOVA) single factor statistical tool indicated that there is a significant difference in the sensitivity of the tested microorganisms to the various extracts (*ρ* < 0.01). The MICs ranged from 0.02 ± 0.00 to 0.52 ± 0.15 mg/ml (Tables 1, 2 and Fig. 2). The microbial sensitivity to the different extracts represented by the mean MIC values ranged from 0.09 to 0.28 mg/ml (Fig. 3). *Escherichiacoli*, a Gram-negative bacterium was as expected from the basis of selection the most sensitive species (MIC = 0.09 mg/ml), followed by *S. aureus* (MIC = 0.20 mg/ml), *S.* Typhimurium (MIC = 0.22 mg/ml) and *E. faecalis* (MIC = 0.28 mg/ml) (Fig. 3).

**Table 2:** Extract yields, Minimal Inhibitory Concentration (MIC) and Total Antibacterial Activity of the nine selected acetone leaf extracts against Gram-positive bacteria

		*Staphylococcus aureus*		*Enterococcus faecalis*		*Bacillus cereus*	
Plants	% yield	MIC (mg/ml)	TAA (ml/g)	MIC (mg/ml)	TAA (ml/g)	MIC (mg/ml)	TAA (ml/g)
*Hypericum roeperianum*	12.0	0.23 ± 0.11	521.59	0.32 ± 0.22	374.90	0.06 ± 0.03	1999.44
*Cremaspora triflora*	2.02	0.02 ± 0.00	1008.33	0.12 ± 0.14	168.06	0.08 ± 0.00	252.08
*Heteromorpha arborescens*	2.60	0.52 ± 0.15	50.06	0.42 ± 0.15	61.98	0.42 ± 0.15	61.98
*Pittosporum viridiflorum*	2.72	0.08 ± 0.00	339.58	0.42 ± 0.15	64.68	0.21 ± 0.07	129.37
*Bolusanthus speciosus*	2.30	0.45 ± 0.26	51.19	0.31 ± 0.00	74.30	0.11 ± 0.04	209.39
*Calpurnia aurea*	2.86	0.12 ± 0.06	238.61	0.21 ± 0.07	136.35	0.11 ± 0.04	260.30
*Maesa lanceolata*	11.12	0.06 ± 0.03	1853.89	0.13 ± 0.13	855.64	0.12 ± 0.14	926.94
*Elaeodendron croceum*	9.00	0.23 ± 0.11	391.16	0.42 ± 0.15	214.21	0.21 ± 0.07	428.41
*Morus mesozygia*	1.85	0.08 ± 0.00	230.83	0.16 ± 0.11	115.42	0.16 ± 0.11	115.42
Gentamicin	NA	0.002	NA	0.0016	NA	0.0002	NA
Average for extracts	NA	0.20 ± 0.17	NA	0.28 ± 0.12	NA	0.12 ± 0.04	NA

*NA* not applicable

The overall sensitivity of *E.coli* confirmed the activities obtained in previous screening against the *E.coli* [13]. Also, Makhafola and Eloff [26] in their preliminary investigation of the antibacterial activity of crude acetone extracts of *Ochna* spp. reported that *E.coli* was the most sensitive bacterial species amongst the tested bacteria. In this study, the mean MIC values of the extracts against *E. faecalis* and *S.* Typhimurium were statistically significantly higher than the MIC value of the extracts against *E. coli* (*ρ* < 0.01) (Fig. 3). Nkuo-Akenj et al. (2001) in Aliero and Ibrahim [27] reported that *S.* Typhimurium was the least sensitive to the extracts of *Commelina bengalensis*, with MIC value of 1 mg/ml, and required a concentration of 4 mg/ml to be bactericidal. The resistance of *S.* Typhimurium is of public health concern, as major salmonellosis outbreaks are caused by the emergence of antibiotic resistant *Salmonella* spp. as a result of the use of antimicrobial growth promoters in animals used as food [27].

Overall, the Gram-negative bacteria were more sensitive to the extracts than the Gram-positive bacteria (Tables 1, 2 and Figs. 2 and 3) but the differences were not statistically significant (*p* > 0.05). These results are in agreement with our earlier findings on compounds isolated from *Ochna* species [28]. The difference in the sensitivity between Gram-negative and Gram-positive bacteria may be due to the variation in their cell wall structure. The Gram-positive bacterial cell wall consists of 70–100 layers of peptidoglycans. Peptidoglycan is comprised of two polysaccharides, *N*-acetyl-glucosamine and *N*-acetyl-muramic acid cross-linked by peptide side chains and cross bridges. This is certainly an oversimplification as an explanation and other mechanisms probably play a role. Resistance from Gram-negative bacteria against antibiotics like penicillin originates from the secretion of the lactamase enzyme in the periplasmic space between the thin outer membrane and the cytoplasmic membrane [29].

**Fig. 4:**
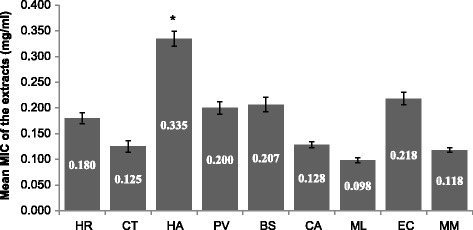
The mean MIC in mg/mlof the acetone leaf extracts of nine plants against all the test bacteria (*ρ* < 0.05). HR = *Hypericum roeperianum*, CT = *Cremaspora triflora*, HA = *Heteromorpha arborescens*, PV = *Pittosporum viridiflorum*, BS = *Bolusanthus speciosus,* CA = *Calpurnia aurea*, ML = *Maesa lanceolata*, EC = *Elaeodendron croceum*, MM = *Morus mesozygia,* ^*^ = mean MIC value of *H. arborescens* is significantly higher than MIC of *M. lanceolata* (*ρ* < 0.01)

The mean difference between the MIC values of the extracts against all tested pathogens was statistically significant (*ρ* < 0.05). The mean MIC of *H. arborescens* (0.335 mg/ml) was significantly higher than the extract of *M. lanceolata* (0.098 mg/ml), *ρ* < 0.01. *Heteromorpha arborescens* was the least active extract and *M. lanceolata* the most potent extract (Fig. 4). *Maesa lanceolata*, *M. mesozygia* and *C. aurea* had good activity against Gram-negative bacteria, mean MIC = 0.09 ± 0.06, 0.10 ± 0.05 and 0.11 ± 0.06 mg/ml respectively. *Cremaspora triflora, M. lanceolata and M. mesozygia* were more potent against all the Gram-positive bacteria, mean MIC values = 0.07 ± 0.05, 0.10 ± 0.04 and 0.13 ± 0.05 mg/ml respectively. *Maesa lanceolat*a extracts had the same MIC value of 0.10 mg/ml against all pathogens making it the most active extract (Fig. 2). Adamu et al. [25] determined the antibacterial and antioxidant activities of 13 South African plants extracts. They also found that *Maesa lanceolata* extracts had higher activity against four nosocomial bacteria than other plant extracts.

The average MIC of the different extracts against *Staphylococcus aureus* was 0.2 mg/ml, with a standard deviation of 0.17 (Table 2). There was a wide range of MICs amongst the different extracts against *Staphylococcus aureus. Cremaspora triflora* had the lowest MIC of 0.02 mg/ml, while *Heteromorpha arborescens* had a high MIC of 0.52 mg/ml. In all, there was a no statistically significant difference in average MIC of the different extracts against all tested Gram-positive bacteria (Table 2). The extracts had lower average MICs values against all tested Gram-negative bacteria compared with Gram-positive bacteria. The extracts had an average MIC of 0.09 mg/ml against *E. coli* and a higher MIC value of 0.22 mg/ml against *Salmonella* Typhimurium. The standard deviation of the mean MIC values of the different extracts was low compared to that of the Gram-positive bacteria. *Cremaspora triflora* extracts had good MIC values of 0.02 mg/ml and 0.05 mg/ml against both *Staphylococcus aureus* (Gram-positive) and *Escherichia coli* (Gram-negative) respectively. The average MIC value of the different extracts against Gram-negative and Gram-positive bacteria was 0.14 mg/ml and 0.21 mg/ml respectively. Standard deviation of the means was 0.04, and differences were not statistically significant. It may be interpreted that the different extracts have similar activity against both Gram-negative and Gram-positive bacteria, giving them a broader spectrum.

**Fig. 5:**
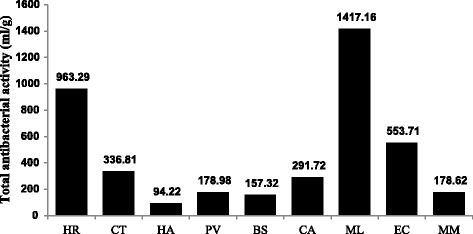
Efficacy (mean TAA values, ml/g) of the different acetone extracts against all the test bacteria. The higher the TAA value, the more efficacious the plant. The quantity extracted from 1 g of CL can be diluted to 1417 ml and will still inhibit on average the different bacteria. HR = *Hypericum roeperianum*, CT = *Cremaspora triflora*, HA = *Heteromorpha arborescens*, PV = *Pittosporum viridiflorum*, BS = *Bolusanthus speciosus,* CA = *Calpurnia aurea*, ML = *Maesa lanceolata*, EC = *Elaeodendron croceum*, MM = *Morus mesozygia*

The total antibacterial activity (TAA), is a function of the extraction yield in milligram per 1 gram of plant material and the minimal inhibitory concentration (MIC), expressed in millilitre per gram (ml/g) [30]. TAA indicates the volume of water or solvent, when added to 1 gram of the extract that will still inhibit the growth of the pathogen [30]. There was a statistically significant difference between the efficacy of the extracts against the tested microorganisms (*ρ* < 0.01). *Maesa lanceolata*, *H. roeperianum* and *E. croceum* had higher activities in the antibacterial assay, with TAA values of 1417, 963 and 554 ml/g respectively (Fig. 5). The MIC and TAA values are useful pharmacological tools in determining the activity of extracts in mg/ml (potency) of plants extracts for isolating bioactive compounds and total activity on ml/g (efficacy) is useful for the selection of plant species [30].

### Cytotoxicity and safety of the extracts

To assume that crude plant extracts or natural products are safe for use could be misleading and dangerous [28]. There were differences in the cytotoxicity of the different extracts (Table 3). *Heteromorpha arborescens* extracts had the lowest toxicity with LC_50_ = 81.0 ± 7.6 μg/ml, followed by *H. roeperianum* (66.2 ± 0.02 μg/ml) and *C. triflora* (57.4 ± 2.94 μg/ml). Plant extracts with LC_50_ ≤ 20 μg/ml are considered toxic [31]. *M. lanceolata* had the lowest LC_50_ at 2.38 ± 0.25 μg/ml, and was even more toxic than the reference drug doxorubicin at 8.3 ± 1.76 μg/ml [32]. Our report on the cytotoxicity of *M. lanceolata* agrees with the reports of Adamu et al. [33] and Muhammad et al. [34]. In addition, crude saponins isolated from the stem bark of *M. lanceolata* are toxic to snails at a concentration of 1 μg/ml and potentially harmful to aquatic biota [35]. Plant extracts that are more toxic to the cells than to bacteria may have no therapeutic value. It is also possible that the cytotoxicity may be due to a general metabolic toxin affecting microorganisms and animal cells. [28]. However, it might be erroneous to draw a conclusion on the cytotoxicity and usefulness of a plant extract by using one or even several cell lines. The toxicity should also be determined in in vivo studies before a definitive conclusion can be reached. Discarding a crude extract because of initial cytotoxic effects on cell lines might be unproductive. The cytotoxic compound may not necessarily be the antibacterial compound. It is at least theoretically possible to isolate a potent, non-toxic novel metabolite from toxic crude plant extracts.

**Table 3:** Cytotoxicity against Vero cells LC_50_ (μg/ml) from Elisha et al., [32] and Selectivity Index of the nine selected acetone crude extracts

	Selectivity index							
Plants	Cytotoxicity	*S. aureus*	*E. faecalis*	*B. cereus*	*E. coli*	*P. aeruginosa*	*S. typhimurium*	Average
*Hypericum roeperianum*	66.2 ± 0.02	0.29	0.21	1.10	0.51	0.83	0.25	0.53
*Cremaspora triflora*	57.4 ± 2.94	2.87	0.48	0.72	1.15	0.36	0.18	0.96
*Heteromorpha arborescens*	81.0 ± 7.6	0.16	0.19	0.19	0.45	0.51	0.26	0.29
*Pittosporum viridiflorum*	54.6 ± 14.3	0.68	0.13	0.26	0.50	0.34	0.25	0.36
*Bolusanthus speciosus*	52.8 ± 3.92	0.12	0.17	0.48	0.66	0.33	0.41	0.36
*Calpurnia aurea*	13.6 ± 2.26	0.11	0.06	0.12	0.34	0.09	0.10	0.14
*Maesa lanceolata*	2.38 ± 0.25	0.04	0.02	0.02	0.06	0.03	0.01	0.03
*Elaeodendron croceum*	5.2 ± 0.24	0.02	0.01	0.02	0.05	0.07	0.02	0.03
*Morus mesozygia*	40.7 ± 1.54	0.51	0.25	0.25	0.58	0.51	0.25	0.39
Doxorubicin	8.3 ± 1.76	ND	ND	ND	ND	ND	ND	ND

*ND* not determined

The selectivity index (SI) is calculated by dividing the LC_50_ in mg/ml by the MIC in mg/ml of the test bacteria. The higher the SI value the safer the extracts may be. Selectivity index values greater than one suggests that extracts are less toxic to the host cell than the bacteria [28]. *Cremaspora triflora* had the best SI against *S. aureus* (2.87) and *E. coli* (1.15) while *H. roeperianum* had SI of 1.15 against *B. cereus* (Table 3). The MIC of the extracts was compared with their cytotoxicity for any noticeable correlation. In all, there was a weak, positive correlation (*R* = 0.45), which was statistically not significant (*ρ* > 0.05). This probably means that in many cases the cytotoxicity was not caused by the same compounds responsible for the antimicrobial activity on both the Gram-positive and Gram-negative bacteria.

## Conclusion

Some of the extracts had a good potential for therapeutic uses against some pathogens. It appears that extracts with high antimicrobial activity against Gram-negative bacteria do not necessarily have high activity against other Gram–negative bacteria compared to Gram-positive bacteria. This may mean that the activity is not related to the differences in cell wall structure. Because there is such a wide range of MICs for different strains of the same bacterial species, it is dangerous to generalize these results for one strain of each of the bacteria although these strains were the strains recommended by the National Committee for Clinical Laboratory Standards to compare different antibiotics.

Further investigation is underway on the two species *C. triflora* and *H. roeperianum* that had promising potency and safety. The potency of many of the extracts on the test bacteria was apparently not due to the presence of a general metabolic toxin but possibly through another mechanism of action. It may be interesting to investigate the mode of action of the extracts against test bacteria and resistant clinical strains.

## Abbreviations

DMSO: Dimethyl Sulphoxide
INT: ρ-iodonotrotetrazolium violet
LC_50_: Lethal concentration (50%)
MH: Müller-Hinton
MIC: Minimum inhibitory concentration
MTT: 3-(4,5-dimethylthiazol-2-yl)-2, 5-diphenyltetrazolium bromide
PBS: Phosphate buffer saline
SD: Standard deviation
SI: Selectivity index
TAA: Total antibacterial activity
